# Subthalamic Nucleus Deep Brain Stimulation: Basic Concepts and Novel Perspectives

**DOI:** 10.1523/ENEURO.0140-17.2017

**Published:** 2017-09-22

**Authors:** Clement Hamani, Gerson Florence, Helmut Heinsen, Birgit R. Plantinga, Yasin Temel, Kamil Uludag, Eduardo Alho, Manoel J. Teixeira, Edson Amaro, Erich T. Fonoff

**Affiliations:** 1 Division of Neurosurgery Sunnybrook Health Sciences Centre, University of Toronto, Toronto, Ontario, Canada; 2 Division of Neuroimaging, Centre for Addiction and Mental Health, Toronto, Ontario, Canada; 3 Department of Radiology, University of São Paulo Medical School, São Paulo, Brazil; 4 Division of Neurosurgery, Department of Neurology, University of São Paulo Medical School, São Paulo, Brazil; 5 Department of Biomedical Image Analysis, Eindhoven University of Technology, Eindhoven, The Netherlands; 6 Department of Neurosurgery, Maastricht University Medical Center, Maastricht, The Netherlands; 7 Donders Institute for Brain, Cognition and Behaviour, Radboud University Medical Center, Nijmegen, The Netherlands; 8 Department of Cognitive Neuroscience, Maastricht University, Maastricht, The Netherlands; 9 Department of Psychiatry, Psychosomatics and Psychotherapy, Center of Mental Health, University Clinic of Würzburg, Würzburg, Germany; 10 Instituto de Ensino e Pesquisa Hospital Sírio-Libanês, São Paulo, Brazil

**Keywords:** subthalamic nucleus, deep brain stimulation, mechanisms, plasticity, anatomy, physiology, neuroimaging

## Abstract

Over the last decades, extensive basic and clinical knowledge has been acquired on the use of subthalamic nucleus (STN) deep brain stimulation (DBS) for Parkinson’s disease (PD). It is now clear that mechanisms involved in the effects of this therapy are far more complex than previously anticipated. At frequencies commonly used in clinical practice, neural elements may be excited or inhibited and novel dynamic states of equilibrium are reached. Electrode contacts used for chronic DBS in PD are placed near the dorsal border of the nucleus, a highly cellular region. DBS may thus exert its effects by modulating these cells, hyperdirect projections from motor cortical areas, afferent and efferent fibers to the motor STN. Advancements in neuroimaging techniques may allow us to identify these structures optimizing surgical targeting. In this review, we provide an update on mechanisms and the neural elements modulated by STN DBS.

## Significance Statement

Over the last decades, extensive basic and clinical knowledge has been acquired on the use of subthalamic nucleus (STN) deep brain stimulation (DBS) for Parkinson’s disease (PD). It is becoming clear that DBS exerts its effects through several mechanisms and influences various neural structures and circuits. In this article, we discuss electrophysiological findings suggesting that stimulation not only modulates activity of neural elements but also leads to novel dynamic states of equilibrium. We also present anatomic data showing that the STN is not a homogeneous structure and review fiber pathways and regions of the nucleus potentially modulated by DBS. Finally, we discuss novel neuroimaging modalities and how these may be used to optimize technical aspects of the surgery.

## Introduction

From its origins to clinical approval, the history of subthalamic nucleus (STN) deep brain stimulation (DBS) for Parkinson’s disease (PD) has been one of extreme success. In the late 1980s, thalamic stimulation was proposed as an alternative to ablative procedures for treating patients with tremor (Benabid et al., 1991). In 1-methyl-4-phenyl-1,2,3,6-tetrahydropyridine (MPTP)-treated nonhuman primates, both STN lesions (Bergman et al., 1990) and stimulation (Benazzouz et al., 1993) were shown to improve parkinsonian features. Soon after, a series of PD patients was successfully treated with STN DBS (Limousin et al., 1995). To date, over 120,000 patients worldwide have been implanted with DBS systems. In PD, marked improvements have been reported in motor symptoms and levodopa-induced involuntary movements (Deuschl et al., 2006; Weaver et al., 2009).

Aside from impacting patient care, investigational data from preclinical models and surgical candidates have yielded significant advancements in our understanding of the physiology and pathophysiology of the basal ganglia. Despite this fact and the 30 years of experience with DBS, its mechanisms of action are still not fully understood.

In this review, we provide an update on mechanisms and structures modulated by STN stimulation. We discuss the complexity of DBS and the fact that neural elements may be excited or inhibited, reaching novel dynamic states of equilibrium. We also review neuroanatomical substrates modulated by DBS in the region of the STN. Finally, we examine how advancements in neuroimaging techniques may allow us to identify specific STN regions, so that this therapy may be optimized.

## Anatomic aspects of the STN and nearby fiber structures

**Figure 1.**
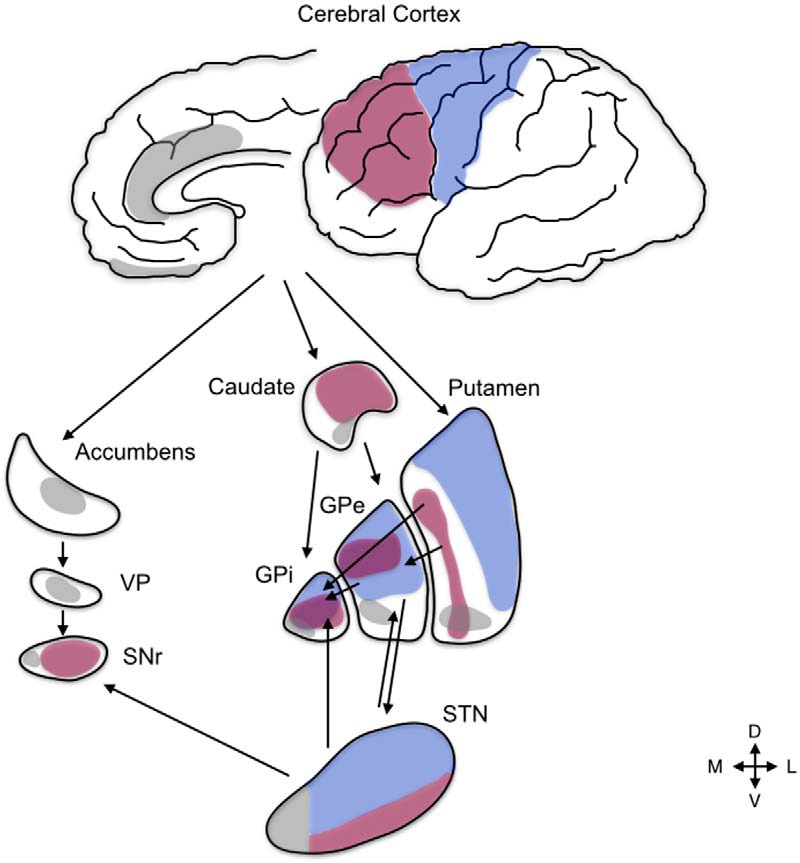
STN and the tripartite model. Intrinsic organization of the STN, basal ganglia structures, and cortical regions according to the tripartite functional subdivision. The motor circuit (blue) includes motor cortical areas (primary motor cortex, supplementary motor cortex, premotor cortex, and portions of the somatosensory dorsal parietal cortex), the dorsolateral portion of the postcommissural putamen, the lateral two-thirds of the globus pallidus (GPe and GPi), and a small portion of the substantia nigra (SNr). In the STN, motor regions comprise dorsal-lateral aspects of the rostrocaudal third of the nucleus (Hamani et al., 2004). Associative circuits (purple) comprise associative cortical regions, most of the caudate nucleus, the putamen rostral to the anterior commissure, the dorsal aspect of the medial third of the globus pallidus (GPe and GPi) and most of the substantia nigra. Associative STN regions may be found in ventral-lateral-rostral portions of the nucleus (Hamani et al., 2004). Limbic circuits (gray) are comprised of limbic cortical areas (e.g., orbitofrontal and the anterior cingulum), the nucleus accumbens and the most rostral portions of the striatum, the subcommissural ventral pallidum (VP), small limbic regions in the ventral portion of the medial third of the globus pallidus (GPe and GPi), the medial tip of the substantia nigra, and the ventral tegmental area. The limbic STN lies in mediorostral portions of the nucleus (Hamani et al., 2004). Arrows represent some of the most important connections between structures. D, dorsal; L, lateral; M, medial; V, ventral. We note that this schematic diagram largely represents structures in two planes with the anteroposterior depiction often lacking. This is the main reason for the superposition of colors representing motor, associative and limbic regions. Parts of this figure were modified and reprinted with permission from Hamani et al. (2004); Krack et al. (2010).

The STN is a lens-shaped densely populated structure, with extensive membrane apposition between the cell bodies, dendrites, and proximal axonal segments (Chang et al., 1983; Afsharpour, 1985; Hamani et al., 2004). It is predominantly composed of glutamatergic projection neurons with 7.5% of cells in humans being identified as GABAergic interneurons (Hamani et al., 2004; Lévesque and Parent, 2005). In primates, the STN has been subdivided in a tripartite arrangement based on physiologic characteristics and the distribution of efferent/afferent projections (Alexander et al., 1990; Parent and Hazrati, 1993, 1995a,b; Hamani et al., 2004; Krack et al., 2010; Fig. 1). The limbic STN and part of the associative territory lie in medial-rostral portions of the nucleus. The ventral-lateral-rostral portion comprises the remainder of the associative region. The dorsolateral aspects of the rostral STN and the caudal third of the nucleus are associated with motor circuits (Parent and Hazrati, 1995a; Shink et al., 1996; Hamani et al., 2004).

## Fiber systems

One of the characteristics of the STN is that it is enveloped by fibers, including the internal capsule, pallidofugal system, and medial lemniscus.

### Pallidofugal systems

**Figure 2.**
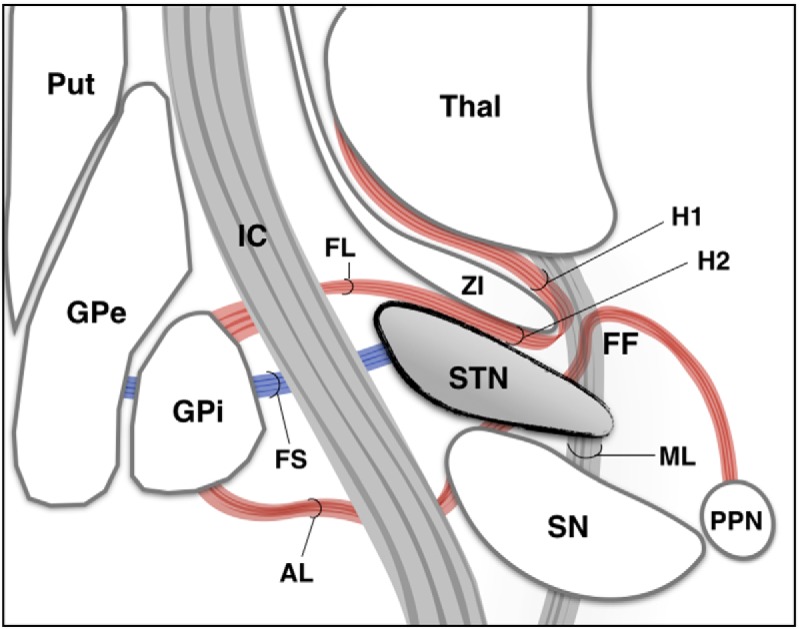
Anatomic aspects of the STN. Principal brain structures surrounding the STN. FF, fields of Forel; FS, subthalamic fascicle; H1, H1 field of Forel (thalamic fasciculus); H2, H2 field of Forel; IC, internal capsule; ML, medial lemniscus; PPN, pedunculopontine nucleus; Put, putamen; SN, substantia nigra; Thal, thalamus; ZI, zona incerta. Part of this figure was modified and reprinted with permission from Hamani et al. (2004).

**Figure 3.**
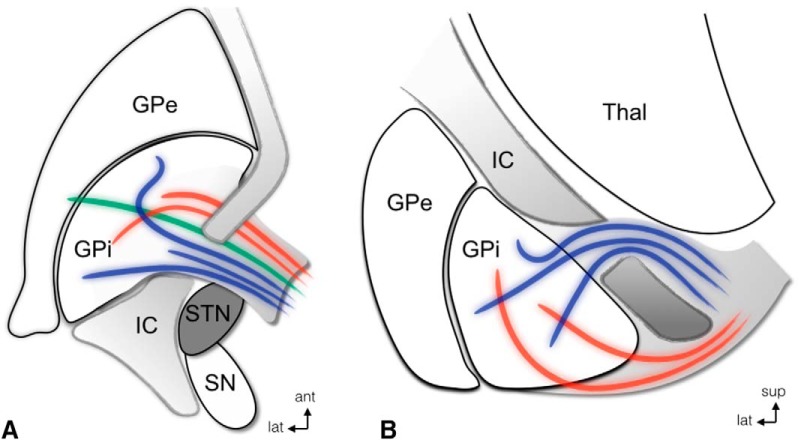
STN and pallidofugal fibers. Axial (***A***) and coronal (***B***) schematic representations of the AL (red) and LF (H2; blue), in relationship to the STN, in nonhuman primates. Note that both the tracts travel dorsal to the most anterior aspect of the STN. ***A***, The thalamic fasciculus is represented in green. ant, anterior; lat, lateral; sup, superior. Modified and reprinted with permission from Parent and Parent (2004).

The ansa lenticularis (AL) and fasciulus lenticularis (FL) are largely comprised by globus pallidus internus (GPi)-thalamic projections. In primates, the former was thought to originate largely from the lateral GPi (Kuo and Carpenter, 1973; Kim et al., 1976), sweeping around the internal capsule and curving posteriorly to reach the H field of Forel (Fig. 2). The FL (H2 field of Forel) was believed to arise from the medial GPi (Kuo and Carpenter, 1973; Kim et al., 1976), perforate the internal capsule, and form a bundle ventral to the zona incerta. In contrast to this classical view, however, recent studies suggest that the AL and FL should be considered as ventral and dorsal portions of a morphologic continuum that harbors pallidofugal axons arising from all sectors of the GPi (Parent and Parent, 2004). Independent of the origin of pallidothalamic projections, the lenticular fasciculus joins the ansa along with fibers from the superior cerebellar peduncle and brainstem in the H field of Forel, forming the thalamic fasciculus (H1 field of Forel; Hamani et al., 2004; Parent and Parent, 2004). An important aspect to be noticed in nonhuman primates is that a substantial portion of pallidofugal fibers seems to run anterior to the motor STN (Fig. 3).

## Afferent and efferent STN projections

### STN-basal ganglia

Projections from the basal ganglia to the STN derive largely from the globus pallidus externus (GPe) via the subthalamic fasciculus, a fiber bundle that enters/departs the STN from its inferolateral border and crosses the internal capsule. Efferents from the STN to the basal ganglia comprise glutamatergic projections that innervate the globus pallidus, substantia nigra and striatum. Although most STN-nigral projections innervate the pars reticulata (SNr), fibers to the pars compacta (SNc) have received considerable attention as a substrate capable of regulating dopamine release (Smith et al., 1990; Parent and Hazrati, 1995a; Rodriguez et al., 1998). Overall, STN-basal ganglia projections seem to follow the tripartite distribution (Fig. 1; Parent and Hazrati, 1995a; Shink et al., 1996; Hamani et al., 2004; Krack et al., 2010).

### STN-cerebral cortex

The hyperdirect pathway is comprised of motor and premotor cortical fibers that travel though the internal capsule and directly innervate the STN. The former innervates the dorsal STN and arises from the primary motor cortex, supplementary motor area (SMA), pre-SMA, as well as the dorsal and ventral premotor cortices (Nambu et al., 1996; Nambu et al., 1997; Nambu et al., 2000). Ventromedial portions of the nucleus receive afferents from the frontal and supplementary frontal eye fields and are involved in circuits related to eye movements (Matsumura et al., 1992). Prefrontal cortical afferents from areas the dorsolateral prefrontal cortex and anterior cingulate cortex terminate in ventromedial and medial regions of the STN, respectively (Haynes and Haber, 2013).

### Thalamus and brainstem

The main projections from the thalamus to the STN originate from the parafascicular and centromedian nuclei (Sadikot et al., 1992; Hamani et al., 2004). Brainstem projections arise from various nuclei and involve multiple neurotransmitter systems. These include dopaminergic fibers from the SNc (Lavoie et al., 1989; François et al., 2000; Hamani et al., 2004), cholinergic and noncholinergic projections from the pedunculopontine nucleus and laterodorsal tegmental nuclei (Carpenter et al., 1981; Mesulam et al., 1992; Lavoie and Parent, 1994), noradrenergic fibers from the locus ceruleus (Carpenter et al., 1981), and serotonergic fibers likely from the raphe (Parent et al., 2011).

## Physiologic properties of the STN and oscillatory activity

STN cells in nonhuman primates fire at 18 ± 25 Hz, mostly in irregular but also regular and bursty patterns (Wichmann et al., 1994a; Hamani et al., 2004). In parkinsonian states, the STN fires more irregularly at higher rates, ultimately disrupting the functioning of downstream basal ganglia structures (Robledo and Féger, 1990; Bergman et al., 1994; Hassani et al., 1996; Hutchison et al., 1998). Also abnormal in PD are cortico-basal ganglia oscillations. STN cells oscillating at frequencies below 10 Hz are sometimes related to parkinsonian tremor (Levy et al., 2000; Magariños-Ascone et al., 2000). Oscillations in the 70- to 85-Hz range occur during movement or treatment with dopaminergic agonists (Magill et al., 2001; Levy et al., 2002; Hamani et al., 2004). Oscillations in the beta range (15–30 Hz) are prominent in sensorimotor regions of the basal ganglia and cortex (Bergman et al., 1994; Brown et al., 2001; Mallet et al., 2008b). These in fact seem to entrain spiking activity in the STN, striatal cholinergic interneurons and basal ganglia downstream structures (Deffains et al., 2016).

In the clinic, while treatment-induced reductions in bradykinesia and rigidity correlate with decreases in beta (Brown et al., 2001; Kühn et al., 2008; Ray et al., 2008; Kühn et al., 2009), STN stimulation at beta frequencies may worsen bradykinesia (Chen et al., 2007; Eusebio et al., 2008). These same results have not been observed in drug-naïve nonhuman primates, which have been shown to develop dystonia and myoclonia but no bradykinesia following STN stimulation (Syed et al., 2012). The actual role of beta oscillations on mechanisms of bradykinesia remains disputed.

Also characteristic of PD are altered cross-frequency interactions (CFIs; López-Azcárate et al., 2010; Shimamoto et al., 2013; de Hemptinne et al., 2015). These are often appreciated when a more complex analysis of interactions between different frequency bands is conducted (Canolty and Knight, 2010). Similar to beta oscillations, CFIs correlate with motor symptoms and may be reversed by the administration of dopaminergic medications (López-Azcárate et al., 2010).

## Behavioral effects of STN stimulation

In rodents, focal injections of GABAergic antagonists into the STN induce postural asymmetry and abnormal movements (Dybdal and Gale, 2000; Périer et al., 2000). Similar to the clinical scenario (Dewey and Jankovic, 1989; Lee and Marsden, 1994), both lesions and the focal inactivation of the STN in nonhuman primates induce ballism, choreic and dyskinetic movements (Hammond et al., 1979; Crossman et al., 1980; Hamada and DeLong, 1992; Beurrier et al., 1997). STN DBS delivered to otherwise naïve nonhuman primates may induce dyskinesias and abnormal movements, particularly when applied at relatively high currents (Beurrier et al., 1997; Hamani et al., 2004). In parkinsonian rodents and primates, STN lesions or high-frequency stimulation (HFS) mitigate motor deficits, bradykinesia, rigidity, and tremor (whenever this is present; Bergman et al., 1990; Aziz et al., 1992; Benazzouz et al., 1993; Wichmann et al., 1994b; Carvalho and Nikkhah, 2001; Darbaky et al., 2003; Hamani et al., 2004).

In addition to motor effects, clinical studies suggest that STN DBS may be associated with impulsivity, cognitive and psychiatric adverse events (Rodriguez-Oroz et al., 2005; Frank et al., 2007; Hälbig et al., 2009; Okun et al., 2009; Weaver et al., 2009; Follett et al., 2010; Bronstein et al., 2011; Rothlind et al., 2015). As patients receiving DBS often have PD and are under pharmacological treatment, the physiologic role of the STN in nonmotor behavior may be better appraised in preclinical models (Hamani and Temel, 2012).

In animals, some of the most commonly investigated nonmotor behaviors are impulsivity, compulsivity, and drug and reward consumption (Hamani and Temel, 2012). Impulsivity can be broadly defined as acting or making decisions without appropriate forethought (Winstanley, 2011). Overall, impulsive behavior encompasses multiple facets, from motor disinhibition to maladaptive decision making, involving motor, attention, and nonplanning aspects (Brunner and Hen, 1997; Evenden, 1999; Winstanley, 2011). Frequently used paradigms to study impulsivity in rodents are those in which individuals need to withhold from making a response (e.g., measurements of reaction time) or have to properly select a response to obtain a reward (e.g., five-choice serial reaction time task; Winstanley, 2011). Commonly observed inappropriate responses during such tasks include prematurely responding to the stimuli or making errors of perseveration. In some of these paradigms, STN lesions or the focal administration of GABAergic agonists in otherwise naïve rats induce impulsive-like behavior (Baunez et al., 1995; Baunez and Robbins, 1997, 1999b). In parkinsonian rodents, STN lesions increase perseverative responses (Baunez and Robbins, 1999a). Compared to lesion studies, the effects of STN DBS are far more controversial. In naïve animals, HFS has been shown not to affect impulse-like behavior (Desbonnet et al., 2004), reduce premature responses (Desbonnet et al., 2004), or even impair performance (e.g., is a visual attention task; Baunez et al., 2007). Similarly, studies in PD animals have shown reversal (Temel et al., 2005; Temel et al., 2006a), no effect (Darbaky et al., 2003), or a transient worsening of associated deficits (Baunez et al., 2007). Reasons for discrepancy across studies remain unclear but may be related to differences in behavioral paradigms, current intensity or the use of unilateral versus bilateral stimulation.

Along with impulsivity, gambling and punding are aspects commonly described as part of the so-called dopamine dysregulation syndrome (DDS; Fenu et al., 2009; O'Sullivan et al., 2009). In the clinic, STN DBS has been used to treat these conditions following the reduction in dopaminergic medication intake (Broen et al., 2011). Preclinical paradigms suited to model some aspects of gambling-type behavior involve the presentation of animals with options associated with variable amounts of reward, from smaller immediate to late but more gratifying compensations (Cocker and Winstanley, 2015). In otherwise naïve rodents, STN-DBS significantly increases the number of premature responses in some of these paradigms (i.e., the selection of immediate disavantageous rewards; Aleksandrova et al., 2013). In contrast, animals bearing STN lesions have a decrease in impulsive decision making and are able to wait for larger delayed rewards (Winstanley et al., 2005; Uslaner and Robinson, 2006).

Another commonly reported side effect of STN DBS is depression (Temel et al., 2006b). Similar to the clinical scenario, rodents treated with STN DBS present depressive-like behavior in different models (Temel et al., 2007; Creed et al., 2013).

In recent years, DBS has been used to treat patients with refractory obsessive-compulsive disorder (OCD; Mallet et al., 2002; Mallet et al., 2008a; Haynes and Mallet, 2010). Preclinical models to mimic this condition are usually characterized by repetitive, excessive and inappropriate behaviors, which may occur either naturally or as a consequence of pharmacological and behavioral manipulations (Joel, 2006; Albelda and Joel, 2012; Hamani and Temel, 2012). A limitation of these paradigms, however, is that they only mimic compulsivity but not obsessions (Albelda and Joel, 2012; Hamani and Temel, 2012). In rodents, STN HFS has been shown to improve perseverative and compulsive-like behaviors (Winter et al., 2008; Klavir et al., 2009). Similarly, nonhuman primates treated with HFS in the limbic portion of the STN had an improvement in compulsive-like features induced the injections of GABAergic antagonists into basal ganglia structures (Baup et al., 2008).

Another interesting aspect of the STN physiology is its role in mechanisms of reward and addiction. In rodents, bilateral STN lesions increase motivation to obtain food reward (Baunez et al., 2002; Baunez et al., 2005; Rouaud et al., 2010) while reducing the preference and willingness to work for cocaine (Baunez et al., 2005; Rouaud et al., 2010). When alcohol is considered, STN lesions increase motivation for drug intake in animals considered to be “high drinkers,” inducing an opposite effect “low drinker” rats (Lardeux and Baunez, 2008). These results highlight the complexity of physiologic mechanisms of the STN on reward.

## Mechanisms of DBS

Single pulses of cathodic extracellular stimulation depolarize cells, axons, and dendrites. Once action potentials are fired, neurons tend to repolarize, and the normal ionic/neurotransmitter baseline equilibrium is reestablished. These same physiologic responses do not occur when stimulation is delivered at clinical frequencies (i.e., 130–185 Hz). For one, only neural appendages fire action potentials in response to HFS. In addition, the continuous delivery of HFS overloads mechanisms responsible for the extracellular removal of certain ions and transmitters (Florence et al., 2016). Ultimately, stimulated regions reach a new dynamic state, characterized by altered ionic currents, nonsynaptic mechanisms, excessive extracellular levels of neurotransmitters/ions (e.g., potassium), and microenvironmental changes that favor the development of plasticity (Hamani and Temel, 2012; Florence et al., 2016).

From a neuronal perspective, a commonly proposed pattern of response following HFS involves the depolarization of axons and functional inhibition of cell bodies (Lozano et al., 2002; Vitek, 2002; Hamani and Temel, 2012; Florence et al., 2016). Although this is well suited to explain some DBS responses, it is rather simplistic. For example, one of the proposed mechanisms for the effects of HFS is the so-called “depolarization block” (Beurrier et al., 2001; Magariños-Ascone et al., 2002; Kringelbach et al., 2007). This has been largely defined as a state in which cells undergo depolarization with an almost complete abolishment of spontaneous action potentials (Beurrier et al., 2001; Magariños-Ascone et al., 2002). The rationale suggesting that depolarization block and a functional target inactivation may play a role in a HFS response stems from the fact that clinical outcome in some DBS applications (e.g., tremor, PD) resembles that observed with lesions. To date, stimulation-induced depolarization blocks have been largely demonstrated in brain slices. *In vivo* studies conducted in rodents (Tai et al., 2003), nonhuman primates (Meissner et al., 2005), and humans (Filali et al., 2004) have shown striking reductions in the firing of STN cells nearby the electrodes. Yet, the mechanisms responsible for this effect may not only involve a depolarization block but also the excitation of pallidal GABAergic terminals to the STN (Filali et al., 2004).

An aspect not commonly reported, however, is that depolarization blocks are not sustainable events. Over time, cells restore repolarizing mechanisms and become once again capable of firing action potentials until the development of a new depolarization block (Zheng et al., 2011; Florence et al., 2016). As a result, the same stimulated region may contain cells that are either functionally blocked or firing in tonic or even bursty modes (Kass and Mintz, 2006; Wu and Shuai, 2012). Also, not commonly described is the fact that cells held in a depolarization block are theoretically capable of releasing neurotransmitters. As the membrane potential becomes more positive and the amplitude of action potentials decreases, both intracellular calcium influx and neurotransmitter release are decreased. Depolarized membranes, however, may still release neurotransmitters in smaller nonquanta amounts, even when the cell stops firing. This “synaptic noise” has in fact been shown to modulate postsynaptic currents (Ammari et al., 2011). Depending on the released neurotransmitter, postsynaptic neurons may depolarize or hyperpolarize, becoming more or less responsive to inputs from other presynaptic cells (Fellous et al., 2003; Faisal et al., 2008). Highlighting the importance of this mechanism, STN synaptic noise has been shown to interrupt abnormal oscillatory patterns in parkinsonian animals (Ammari et al., 2011). That said, further evidence is required to confirm the relevance of synaptic noise-associated neurotransmitter release as a mechanism of DBS.

Another commonly proposed mechanism underlying the effects of HFS is the excitation of fiber pathways (afferent and efferent projections from targeted regions as well as en passant fibers; Kringelbach et al., 2007). This is of importance, as the anterograde and retrograde propagation of action potentials may influence the physiology of brain regions at a distance from the original stimulation site (Windels et al., 2000; Hashimoto et al., 2003; Kringelbach et al., 2007; Temel et al., 2007). Fibers modulated by HFS may be those arriving, departing or passing through (en passant) the target zone. Neurotransmitters released may dictate the effects of DBS at a distance. For example, with a predominance of glutamatergic projection cells DBS in the STN has been shown to increase cell firing in structures innervated by the nucleus (Hashimoto et al., 2003). Microdialysis studies corroborate this assertion, showing glutamate release in output basal ganglia structures (Windels et al., 2000). However, with a complex interplay of modulated afferent and efferent projections, the net effects of DBS are not always predictable. As an example, STN DBS has been shown to significantly reduce neuronal firing in the nigra, particularly when applied at lower amplitudes (Maurice et al., 2003; Tai et al., 2003). This may occur due to an increased release of GABA via the modulation of pallidal activity (Windels et al., 2005). Also contributing to a functional inhibition of circuits, cells at a distance from the DBS target may not recognize stimulation-driven high-frequency rhythms that replace physiologic firing patterns (i.e., “jamming”; Benabid et al., 2002). Finally, we note that, though the main consequence of DBS at 130–185 Hz is to drive axonal projections, frequencies closer to 200 Hz may potentially lead to a state of intermittent excitation or even partial blockage of axonal firing (Kilgore and Bhadra, 2004; Florence et al., 2016).

Also described following HFS are changes in glial activity, synaptic transmission and the development of various forms of plasticity (Hamani et al., 2012; Cooperrider et al., 2014). In some clinical applications (e.g., dystonia, epilepsy) the effects of DBS are often protracted or build up with time. Although an immediate clinical benefit is often appreciated in PD, when batteries expire patients may not present the same preoperative symptoms or medication requirements, suggesting that plastic events may have reorganized the system.

To date, several studies in PD patients and animal models have shown that STN HFS reduces beta oscillations, coherence between motor cortex and STN activity, and phase amplitude coupling (Wingeier et al., 2006; Eusebio et al., 2008; Kühn et al., 2008; Bronte-Stewart et al., 2009; Giannicola et al., 2010; Tass et al., 2012; de Hemptinne et al., 2015). Some of these signals have been recently proposed to feed closed-loop stimulation systems. Studies in nonhuman primates (Rosin et al., 2011; Johnson et al., 2016) and PD patients (Little et al., 2013) have shown that, compared to regular or intermittent HFS, stimulation delivered following the detection of beta oscillatory bursts or according to the pattern of neuronal firing induce a similar or slightly more pronounced clinical improvement. In a recent report, however, closed-loop STN stimulation delivered to PD primates did not improve bradykinesia during a reaching task (Johnson et al., 2016). This result has been attributed to the fact that beta amplitude declines during motion and suggests that additional work is still needed before closed-loop stimulation may be implemented in the clinic.

## Cytoarchitectonic features and elements modulated by DBS

**Figure 4.**
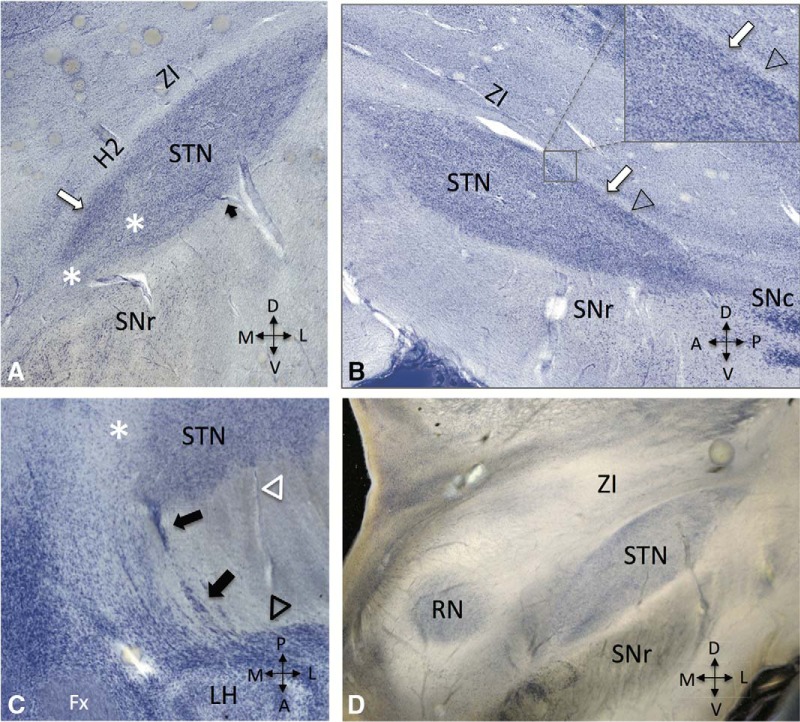
Histologic sections of the subthalamic nucelus (STN) region in individuals with no neurologic disorders stained for gallocyanin. ***A***, Note the presence of high-density cellular regions near the borders of the nucleus (white arrow) and fibers inside its core (*). The dark arrow points to a vessel branching in the vicinity of the STN. ***B***, Sagittal section (400-µm thickness) showing high-density neuronal clusters (white arrow) and a region largely comprised by capsular fibers (arrowhead) near the dorsal border of the STN. Magnified view is presented in the square above. ***C***, Axial (horizontal) section (440-µm thickness) showing the anteromedial aspect of the STN in relation to the lateral hypothalamus (LH) and fornix (Fx). Black arrows denote subthalamic cell strands piercing the internal capsule and forming dissipated accessory cell groups (black open triangle) near the lateral hypothalamus. White open triangles represent the irregular boundary between STN cell clusters and capsular fibers. ***D***, Coronal section (440-µm thickness) showing the STN region under dark-field illumination (RN, red nucleus). SNr, substantia nigra reticulata; SNc, substantia nigra compacta; ZI, zona incerta.

As most human studies addressing cytoarchitectonic features were conducted with classical staining techniques, a few caveats need to be taken into account. First, the STN has an intimate relationship and is partly enwrapped by fibers. Second, its axes are not arranged in parallel to the main axes of the hemispheres. Third, depending on the plane of section, shape and individual orientation, different profiles and grazing artifacts may be observed in Nissl or Golgi-stained sections. Finally, cytoarchitectonic delineations are subject to interindividual variability. When thick gallocyanin stained slices and dark-field illumination sections are examined (Heinsen et al., 2000), a few aspects not previously reported in classical postmortem human studies can be appreciated. (1) Rather than a homogeneous structure, the STN has a looser cellular core and densely packed peripheral regions (Fig. 4). (2) Fiber bundles may be identified near the STN borders as well as in central parts of the nucleus. (3) The medial STN has a fairly irregular outline, with rostromedial strands of cells almost reaching the hypothalamus (Fig. 4).

**Figure 5.**
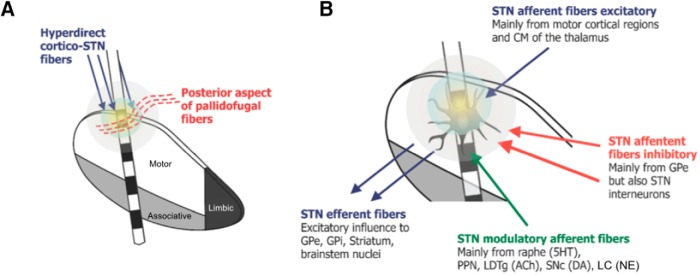
Neural elements modulated by DBS delivered to the dorsal region of the motor subthalamic nucleus (STN) territory. ***A***, Schematic representation of the STN showing potential fiber pathways modulated by DBS. Hyperdirect STN projections from motor cortical regions are depicted in blue. Pallidofugal fibers are depicted in red. ***B***, Schematic representation of an STN neuron modulated by DBS. STN axons driven by stimulation would excite connected structures. Stimulation of STN afferents would potentially excite these projections, inducing complex effects. STN cells would be excited by stimulation of cortical and thalamic-STN projections (blue) and inhibited by stimulation of globus pallidus projections and appendages from local interneurons (red). Stimulation of brainstem-STN projections would modulate STN neuronal activity via different neurotransmitter systems (green). 5HT, serotonin; ACh, acetylcholine; CM, centromedian nucleus of the thalamus; DA, dopamine; GPe, globus pallidus externus; GPi, globus pallidus internus; LC, locus ceruleus; LDTg, laterodorsal tegmental area; NE, norepinephrine; PPN, pedunculopontine nucleus; SNc, substantia nigra compacta. Part of the figure was reprinted with permission from Hamani et al. (2004).

Electrode contacts used for chronic DBS in PD are often located near the dorsal border of the nucleus (Herzog et al., 2004; Pollo et al., 2007). In addition to being part of the motor territory, this region is characterized by the presence of high-density cellular clusters in postmortem studies. The main fiber pathways entering the motor STN are hyperdirect projections from motor cortical areas, which in fact may be a major substrate modulated by STN DBS (Fig. 5). In agreement with this statement, optogenetic studies have shown that stimulation of hyperdirect pathways may rescue behavioral deficits in parkinsonian rodents (Gradinaru et al., 2009). To date, the modulation of fibers in the fields of Forel, particularly pallidothalamic projections, have been proposed as a potential mechanism for the effects of DBS. Although not many studies have reported anatomic details of these systems in humans, data from nonhuman primates suggest that most of the AL and FL lie slightly anterior to the region where electrodes are often implanted. Other substrates that could be potentially modulated by DBS are axons within the motor STN territory. These may comprise afferents/efferents to and from the motor STN. Stimulation of the former would theoretically excite or inhibit the target zone, depending on the neurotransmitter released (e.g., glutamate from cortical/thalamic afferents, GABA from pallidal afferents, serotonin/dopamine/acetylcholine from brainstem afferents). The excitation of STN glutamatergic efferents would drive activity in structures receiving its projections.

DBS electrodes used to treat OCD are placed in anteromedial regions of the STN (Mallet et al., 2008a). Under these circumstances, cell bodies modulated by stimulation would be those innervating limbic/associative STN territories and nearby hypothalamic regions. Hyperdirect components would be fibers from the dorsolateral prefrontal cortex, orbitofrontal cortex and cingulate gyrus. Stimulated afferents/efferents to and from the STN would be those innervating limbic/associative regions of the basal ganglia, thalamus and brainstem. As PD patients who develop DBS-induced psychiatric side effects often have electrodes implanted medially, the same neural elements could be theoretically involved in mechanisms of these adverse events.

## Neuroimaging

**Figure 6.**
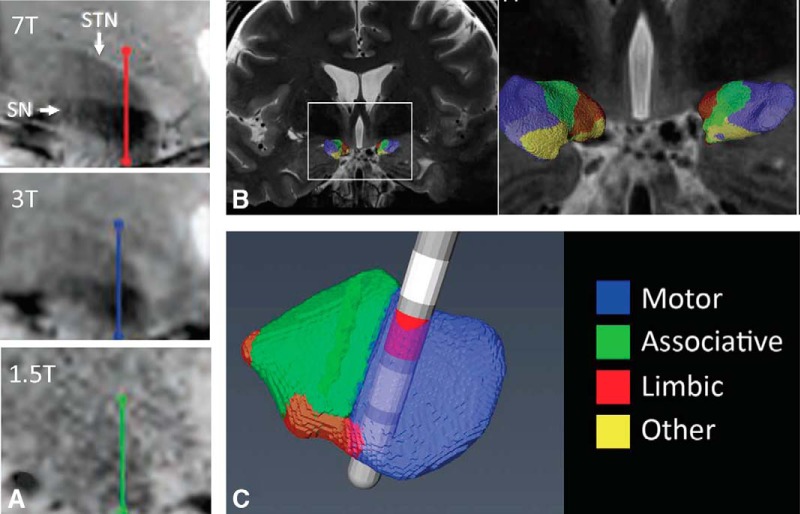
Tractography based subdivision of the STN. ***A***, CoronalT2∗-weighted images obtained at 7.0T, 3.0T, and 1.5T. ***B***, Coronal images showing STN connectivity with limbic (red), associative (green), motor (blue), and remaining (yellow) cortical areas. ***C***, Oblique view of the STN with a superposed DBS electrode and an active contact implanted in the motor territory. Reprinted with permission from Cho et al. (2010); Plantinga et al. (2014); Plantinga et al. (2016).

Adequate visualization of the STN greatly depends on MRI protocols. On T2, T2*, and susceptibility weighted images, the nucleus appears as a dark structure. At 1.5T and less so at 3T, MRI identification of the STN may be hindered by limited imaging contrast and the poor identification of the STN/SN border (Fig. 6). Ultrahigh field (7T and higher) MRI has the potential to overcome some of these limitations and promises to facilitate patient-specific direct targeting (Plantinga et al., 2014).

As described above, the STN may be subdivided in different subterritories based anatomic connections and physiologic characteristics. The use of diffusion weighted imaging based tractography has been proposed as a potential strategy for classifying deep brain structures into subregions (Behrens et al., 2003). The simplest of these models is diffusion tensor, which can be created with relatively short scan times but fails when there are multiple fiber orientations within one voxel. More advanced models seem to be able to cope with crossing fibers when combined with probabilistic tracking algorithms (Behrens et al., 2007; Tournier et al., 2007). On a group level, these models have been used to subdivide the STN into functional regions in 3T scanners (Lambert et al., 2012). At 7T, the motor region could be successfully discriminated based on structural connectivity (Plantinga et al., 2016; Fig. 6). Although this technique is not without limitations (e.g., false positives and negatives), these results highlight the potential future application of neuroimaging strategies to refine surgical targeting.

## Conclusions

In summary, the mechanisms involved in the effects of DBS seem to be far more complex than previously anticipated. Instead of a simple excitation of fibers and inhibition of cells, neural elements influenced by DBS reach novel dynamic states over time. From an anatomic perspective, human pathologic specimens suggest that the STN has dense cellular aggregates near its borders and a less compact central core, which is infiltrated by fibers. Discriminating the nature of these fibers and those crossing the dorsal STN border (i.e., where active contacts are implanted) will be crucial for a better appraisal of mechanisms responsible for this therapy.

One of the ultimate goals to be achieved with DBS is to maximize efficacy while minimizing side effects. The former has been attempted by mimicking brain rhythms so that some forms of beta band activity and other pathologic rhythms may be reduced. To date, similar strategies have been effective in preclinical models but still need to be perfected for clinical use. With proven efficacy, a key factor to minimize DBS-induced side effects is to avoid stimulating structures and brain regions involved in adverse events. A major advance toward this objective is the use of directional leads, which may deviate and steer current away from these structures. Also important have been recent advancements in neuroimaging modalities. The use of higher magnetic fields and diffusion/connectivity approaches to identify subregions of the nucleus and specific fiber bundles may advance the way we do surgery by improving targeting precision.

## Synthesis

Reviewing Editor: Christian Lüscher, University of Geneva Decisions are customarily a result of the Reviewing Editor and the peer reviewers coming together and discussing their recommendations until a consensus is reached. When revisions are invited, a fact-based synthesis statement explaining their decision and outlining what is needed to prepare a revision will be listed below. The following reviewer(s) agreed to reveal their identity: Abdelhamid Benazzouz, Peter Brown

The review has new been seem by two experts, who agree that a major revision is required.

Basic concepts of subthalamic deep brain stimulation are reviewed but the overall organization of the ms needs to be improved. I suggest starting with the anatomy (cell types and connections), then the physiological and pathological activity of the neurons (careful not to mix single cell activity with LFP/Oscillations) to then ask what high frequency stimulation does to the STN. I would also be interesting to read that the authors believe what an ideal stimulation should do.

I also strongly encourage the authors to rework the figures and generate novel original schematics rather than reproducing existing ones.

Finally I attach the comments of the two referees, with their suggestions, which I fully support.

REF1

Major comments :

1) The authors are very selective in the citations and this negatively influences the quality of the review.

2) A major problem is the lack of linkage between the different parts of the review making the order of these different parts not logical and unjustified.

Minor comments :

- Line 27

Replace « the STN in not » by « the STN is not »

- The introduction of the review is very short without any reference. It is mandatory that the authors start with the princeps studies focusing on the mechanisms of STN DBS who showed the inhibitory action of STN DBS on STN neurons and also on the cells of the output nuclei of the basal ganglia receiving the afferent projections from the STN.

- Page 3 : When discussing the phenomenon of depolarization block, the authors mentioned only in vitro studies on slices. It would be very important to cite also in vivo studies in the rat especially Tai et al. (2003), who showed that STN DBS induced a total inhibition of STN neurons, which can be assimilated to a depolarization block effect, in only a small number of STN neurons and that the majority of cells showed a decrease in the firing rate without cell silencing. Another study in non-human primate should be cited (Meissner et al., 2005).

- Page 4, parag start line 77

The authors should also mention references of works showing that STN HFS induced inhibitory effect on the SNr, which is an efferent structure of the STN (Maurice et al., 2003; Tai et al., 2003).

- Page 5, line 110:

The authors mentioned that STN stimulation at beta frequencies worsens bradykinesia. However, they did not mention that STN stimulation using beta frequency oscillations in normal monkeys did not induce bradykinesia nor rigidity (Syed et al., 2012). This demonstrates that beta oscillations are not causal for these symptoms. This should be discussed.

- Page 8, line 181:

In this paragraph the noradrenergic proections from the locus coeruleus to the STN should also be mentioned.

- Page 11, line 250:

The figure 5 mentioned in the manuscript is not attached.

REF2

I would encourage the authors though to either come up with a provisional synthesis of the reviewed data or end with a set of hypotheses to be tested or questions needing pressing answers. In addition, although the review is strong on the connectivity and structure of the STN and borders, the relevance of these observations to the mechanisms of DBS is under worked. In a similar vein, it is difficult to understand what STN DBS may achieve, both in terms of improvement and side-effects, without considering the possible functions of the STN in behaviour first.

Minor comments

1. Some core observations regarding the mechanisms of DBS need more detail eg p3 lines 47-51.

2. P3 second para. I am not sure that the potential dynamic nature of depolarisation block obviates this as a mechanism of action at the population level.

3. P4 top para. I am not sure that the ‘synaptic noise’ hypothesis rules out depolarisation block as a potential mechanism either. Questions remain about the quantitative importance of synaptic noise and as neurotransmitter release under these circumstances is not triggered by action potentials in afferents it is questionable whether such activity caries any information.

4. Lines 82-85 on p 4are not well expressed. I think the authors mean that the effect of DBS on fibres is excitatory per se but that some of these fibres may then release inhibitory neurotransmitters.

5. P5 top. It is stated that patients with PD do not immediately deteriorate once batteries expire. This is certainly not uniformly the case, and the sudden and severe deterioration can present a medical emergency.

6. P5 line 104. 70 to 85 Hz range and not 70{plus minus}85 Hz.

7. P5 bottom, non-specialist readers may need some explanation as to what is meant by ‘altered cross-frequency interactions’.

8. P7 subtitle ‘STN projections’ might be better expressed as projections to and from the STN to cover the material presented.

9. Figure 3 is rather optimistic in the small size of the ‘sphere’ of electrical effect, and with respect to the relative dimeter of the electrode.

10. The review does not mention a key recent paper establishing the key role of the subthalamic nucleus in basal ganglia physiology and pathophysiology that helps explain why this nucleus is such an effective site for invasive treatment (Deffains et al, Elife 2016).

## References

[B1] Afsharpour S (1985) Light microscopic analysis of Golgi-impregnated rat subthalamic neurons. J Comp Neur 236:1–13. 10.1002/cne.902360102 4056088

[B2] Albelda N, Joel D (2012) Animal models of obsessive-compulsive disorder: exploring pharmacology and neural substrates. Neurosci Biobehav Rev 36:47–63. 10.1016/j.neubiorev.2011.04.006 21527287

[B3] Aleksandrova LR, Creed MC, Fletcher PJ, Lobo DS, Hamani C, Nobrega JN (2013) Deep brain stimulation of the subthalamic nucleus increases premature responding in a rat gambling task. Behav Brain Res 245:76–82. 10.1016/j.bbr.2013.02.011 23434606

[B4] Alexander GE, Crutcher MD, DeLong MR (1990) Basal ganglia-thalamocortical circuits: parallel substrates for motor, oculomotor, "prefrontal" and "limbic" functions. Prog Brain Res 85:119–146. 2094891

[B5] Ammari R, Bioulac B, Garcia L, Hammond C (2011) The subthalamic nucleus becomes a generator of bursts in the dopamine-depleted state. Its high frequency stimulation dramatically weakens transmission to the globus pallidus. Front Syst Neurosci 5:43. 10.3389/fnsys.2011.00043 21716635 PMC3115486

[B6] Aziz TZ, Peggs D, Agarwal E, Sambrook MA, Crossman AR (1992) Subthalamic nucleotomy alleviates parkinsonism in the 1-methyl-4-phenyl-1,2,3,6-tetrahydropyridine (MPTP)-exposed primate. Br J Neurosurg 6:575–582. 1361741 10.3109/02688699209002375

[B7] Baunez C, Robbins TW (1997) Bilateral lesions of the subthalamic nucleus induce multiple deficits in an attentional task in rats. Eur J Neurosci 9:2086–2099. 10.1111/j.1460-9568.1997.tb01376.x 9421169

[B8] Baunez C, Robbins TW (1999a) Effects of dopamine depletion of the dorsal striatum and further interaction with subthalamic nucleus lesions in an attentional task in the rat. Neuroscience 92:1343–1356. 10426489 10.1016/s0306-4522(99)00065-2

[B9] Baunez C, Robbins TW (1999b) Effects of transient inactivation of the subthalamic nucleus by local muscimol and APV infusions on performance on the five-choice serial reaction time task in rats. Psychopharmacology (Berl) 141:57–65. 9952065 10.1007/s002130050806

[B10] Baunez C, Nieoullon A, Amalric M (1995) In a rat model of parkinsonism, lesions of the subthalamic nucleus reverse increases of reaction time but induce a dramatic premature responding deficit. J Neurosci 15:6531–6541. 7472415 10.1523/JNEUROSCI.15-10-06531.1995 PMC6578020

[B11] Baunez C, Amalric M, Robbins TW (2002) Enhanced food-related motivation after bilateral lesions of the subthalamic nucleus. J Neurosci 22:562–568. 11784803 10.1523/JNEUROSCI.22-02-00562.2002 PMC6758660

[B12] Baunez C, Dias C, Cador M, Amalric M (2005) The subthalamic nucleus exerts opposite control on cocaine and ‘natural’ rewards. Nat Neurosci 8:484–489. 10.1038/nn1429 15793577

[B13] Baunez C, Christakou A, Chudasama Y, Forni C, Robbins TW (2007) Bilateral high-frequency stimulation of the subthalamic nucleus on attentional performance: transient deleterious effects and enhanced motivation in both intact and parkinsonian rats. Eur J Neurosci 25:1187–1194. 10.1111/j.1460-9568.2007.05373.x 17331214 PMC1877866

[B14] Baup N, Grabli D, Karachi C, Mounayar S, François C, Yelnik J, Féger J, Tremblay L (2008) High-frequency stimulation of the anterior subthalamic nucleus reduces stereotyped behaviors in primates. J Neurosci 28:8785–8788. 10.1523/JNEUROSCI.2384-08.2008 18753380 PMC6670823

[B15] Behrens TE, Johansen-Berg H, Woolrich MW, Smith SM, Wheeler-Kingshott CA, Boulby PA, Barker GJ, Sillery EL, Sheehan K, Ciccarelli O, Thompson AJ, Brady JM, Matthews PM (2003) Non-invasive mapping of connections between human thalamus and cortex using diffusion imaging. Nat Neurosci 6:750–757. 10.1038/nn1075 12808459

[B16] Behrens TE, Berg HJ, Jbabdi S, Rushworth MF, Woolrich MW (2007) Probabilistic diffusion tractography with multiple fibre orientations: what can we gain? Neuroimage 34:144–155. 10.1016/j.neuroimage.2006.09.018 17070705 PMC7116582

[B17] Benabid AL, Pollak P, Gervason C, Hoffmann D, Gao DM, Hommel M, Perret JE, de Rougemont J (1991) Long-term suppression of tremor by chronic stimulation of the ventral intermediate thalamic nucleus. Lancet 337:403–406. 1671433 10.1016/0140-6736(91)91175-t

[B18] Benabid AL, Benazzous A, Pollak P (2002) Mechanisms of deep brain stimulation. Mov Disord 17 [Suppl 3]:S73–S74. 10.1002/mds.10145 11948758

[B19] Benazzouz A, Gross C, Féger J, Boraud T, Bioulac B (1993) Reversal of rigidity and improvement in motor performance by subthalamic high-frequency stimulation in MPTP-treated monkeys. Eur J Neurosci 5:382–389. 8261116 10.1111/j.1460-9568.1993.tb00505.x

[B20] Bergman H, Wichmann T, DeLong MR (1990) Reversal of experimental parkinsonism by lesions of the subthalamic nucleus. Science 249:1436–1438. 2402638 10.1126/science.2402638

[B21] Bergman H, Wichmann T, Karmon B, DeLong MR (1994) The primate subthalamic nucleus. II. Neuronal activity in the MPTP model of parkinsonism. J Neurophysiol 72:507–520. 7983515 10.1152/jn.1994.72.2.507

[B22] Beurrier C, Bezard E, Bioulac B, Gross C (1997) Subthalamic stimulation elicits hemiballismus in normal monkey. Neuroreport 8:1625–1629. 9189903 10.1097/00001756-199705060-00014

[B23] Beurrier C, Bioulac B, Audin J, Hammond C (2001) High-frequency stimulation produces a transient blockade of voltage-gated currents in subthalamic neurons. J Neurophysiol 85:1351–1356. 11287459 10.1152/jn.2001.85.4.1351

[B24] Broen M, Duits A, Visser-Vandewalle V, Temel Y, Winogrodzka A (2011) Impulse control and related disorders in Parkinson's disease patients treated with bilateral subthalamic nucleus stimulation: a review. Parkinsonism Relat Disord 17:413–417. 10.1016/j.parkreldis.2011.02.013 21382739

[B25] Bronstein JM, Tagliati M, Alterman RL, Lozano AM, Volkmann J, Stefani A, Horak FB, Okun MS, Foote KD, Krack P, Pahwa R, Henderson JM, Hariz MI, Bakay RA, Rezai A, Marks WJ Jr, Moro E, Vitek JL, Weaver FM, Gross RE, et al. (2011) Deep brain stimulation for Parkinson disease: an expert consensus and review of key issues. Arch Neurol 68:165. 10.1001/archneurol.2010.260 20937936 PMC4523130

[B26] Bronte-Stewart H, Barberini C, Koop MM, Hill BC, Henderson JM, Wingeier B (2009) The STN beta-band profile in Parkinson's disease is stationary and shows prolonged attenuation after deep brain stimulation. Exp Neurol 215:20–28. 10.1016/j.expneurol.2008.09.008 18929561

[B27] Brown P, Oliviero A, Mazzone P, Insola A, Tonali P, Di Lazzaro V (2001) Dopamine dependency of oscillations between subthalamic nucleus and pallidum in Parkinson's disease. J Neurosci 21:1033–1038. 11157088 10.1523/JNEUROSCI.21-03-01033.2001 PMC6762327

[B28] Brunner D, Hen R (1997) Insights into the neurobiology of impulsive behavior from serotonin receptor knockout mice. Ann NY Acad Sci 836:81–105. 9616795 10.1111/j.1749-6632.1997.tb52356.x

[B29] Canolty RT, Knight RT (2010) The functional role of cross-frequency coupling. Trends Cogn Sci 14:506–515. 10.1016/j.tics.2010.09.001 20932795 PMC3359652

[B30] Carpenter MB, Carleton SC, Keller JT, Conte P (1981) Connections of the subthalamic nucleus in the monkey. Brain Res 224:1–29. 7284825 10.1016/0006-8993(81)91113-6

[B31] Carvalho GA, Nikkhah G (2001) Subthalamic nucleus lesions are neuroprotective against terminal 6-OHDA-induced striatal lesions and restore postural balancing reactions. Exp Neurol 171:405–417. 10.1006/exnr.2001.7742 11573992

[B32] Chang HT, Kita H, Kitai ST (1983) The fine structure of the rat subthalamic nucleus: an electron microscopic study. J Comp Neur 221:113–123. 10.1002/cne.902210110 6643743

[B33] Chen CC, Litvak V, Gilbertson T, Kühn A, Lu CS, Lee ST, Tsai CH, Tisch S, Limousin P, Hariz M, Brown P (2007) Excessive synchronization of basal ganglia neurons at 20 Hz slows movement in Parkinson's disease. Exp Neurol 205:214–221. 10.1016/j.expneurol.2007.01.027 17335810

[B34] Cho ZH, Min HK, Oh SH, Han JY, Park CW, Chi JG, Kim YB, Paek SH, Lozano AM, Lee KH (2010) Direct visualization of deep brain stimulation targets in Parkinson disease with the use of 7-tesla magnetic resonance imaging. J Neurosurg 113:639–647. 10.3171/2010.3.JNS091385 20380532 PMC3160785

[B35] Cocker PJ, Winstanley CA (2015) Irrational beliefs, biases and gambling: exploring the role of animal models in elucidating vulnerabilities for the development of pathological gambling. Behav Brain Res 279:259–273. 10.1016/j.bbr.2014.10.043 25446745

[B36] Cooperrider J, Furmaga H, Plow E, Park HJ, Chen Z, Kidd G, Baker KB, Gale JT, Machado AG (2014) Chronic deep cerebellar stimulation promotes long-term potentiation, microstructural plasticity, and reorganization of perilesional cortical representation in a rodent model. J Neurosci 34:9040–9050. 10.1523/JNEUROSCI.0953-14.2014 24990924 PMC4078081

[B37] Creed MC, Hamani C, Nobrega JN (2013) Effects of repeated deep brain stimulation on depressive- and anxiety-like behavior in rats: comparing entopeduncular and subthalamic nuclei. Brain Stimul 6:506–514. 10.1016/j.brs.2012.09.012 23088853

[B38] Crossman AR, Sambrook MA, Jackson A (1980) Experimental hemiballismus in the baboon produced by injection of a gamma-aminobutyric acid antagonist into the basal ganglia. Neurosci Lett 20:369–372. 7443083 10.1016/0304-3940(80)90176-7

[B39] Darbaky Y, Forni C, Amalric M, Baunez C (2003) High frequency stimulation of the subthalamic nucleus has beneficial antiparkinsonian effects on motor functions in rats, but less efficiency in a choice reaction time task. Eur J Neurosci 18:951–956. 10.1046/j.1460-9568.2003.02803.x 12925021

[B40] de Hemptinne C, Swann NC, Ostrem JL, Ryapolova-Webb ES, San Luciano M, Galifianakis NB, Starr PA (2015) Therapeutic deep brain stimulation reduces cortical phase-amplitude coupling in Parkinson's disease. Nat Neurosci 18:779–786. 10.1038/nn.3997 25867121 PMC4414895

[B41] Deffains M, Iskhakova L, Katabi S, Haber SN, Israel Z, Bergman H (2016) Subthalamic, not striatal, activity correlates with basal ganglia downstream activity in normal and parkinsonian monkeys. Elife 5 10.7554/eLife.16443 PMC5030093 27552049

[B42] Desbonnet L, Temel Y, Visser-Vandewalle V, Blokland A, Hornikx V, Steinbusch HW (2004) Premature responding following bilateral stimulation of the rat subthalamic nucleus is amplitude and frequency dependent. Brain Res 1008:198–204. 10.1016/j.brainres.2004.02.032 15145757

[B43] Deuschl G, Schade-Brittinger C, Krack P, Volkmann J, Schäfer H, Bötzel K, Daniels C, Deutschländer A, Dillmann U, Eisner W, Gruber D, Hamel W, Herzog J, Hilker R, Klebe S, Kloss M, Koy J, Krause M, Kupsch A, Lorenz D, et al. (2006) A randomized trial of deep-brain stimulation for Parkinson's disease. N Engl J Med 355:896–908. 10.1056/NEJMoa060281 16943402

[B44] Dewey RB Jr, Jankovic J (1989) Hemiballism-hemichorea. Clinical and pharmacologic findings in 21 patients. Arch Neurol 46:862–867. 2757526 10.1001/archneur.1989.00520440044020

[B45] Dybdal D, Gale K (2000) Postural and anticonvulsant effects of inhibition of the rat subthalamic nucleus. J Neurosci 20:6728–6733. 10964979 10.1523/JNEUROSCI.20-17-06728.2000 PMC6772946

[B46] Eusebio A, Chen CC, Lu CS, Lee ST, Tsai CH, Limousin P, Hariz M, Brown P (2008) Effects of low-frequency stimulation of the subthalamic nucleus on movement in Parkinson's disease. Exp Neurol 209:125–130. 10.1016/j.expneurol.2007.09.007 17950279 PMC2288636

[B47] Evenden J (1999) Impulsivity: a discussion of clinical and experimental findings. J Psychopharmacol 13:180–192. 10.1177/026988119901300211 10475725

[B48] Faisal AA, Selen LP, Wolpert DM (2008) Noise in the nervous system. Nat Rev Neurosci 9:292–303. 10.1038/nrn2258 18319728 PMC2631351

[B49] Fellous JM, Rudolph M, Destexhe A, Sejnowski TJ (2003) Synaptic background noise controls the input/output characteristics of single cells in an *in vitro* model of in vivo activity. Neuroscience 122:811–829. 10.1016/j.neuroscience.2003.08.027 14622924 PMC2928821

[B50] Fenu S, Wardas J, Morelli M (2009) Impulse control disorders and dopamine dysregulation syndrome associated with dopamine agonist therapy in Parkinson's disease. Behav Pharmacol 20:363–379. 10.1097/FBP.0b013e32833109a0 19724195

[B51] Filali M, Hutchison WD, Palter VN, Lozano AM, Dostrovsky JO (2004) Stimulation-induced inhibition of neuronal firing in human subthalamic nucleus. Exp Brain Res 156:274–281. 10.1007/s00221-003-1784-y 14745464

[B52] Florence G, Sameshima K, Fonoff ET, Hamani C (2016) Deep brain stimulation: more complex than the inhibition of cells and excitation of fibers. Neuroscientist 22:332–345. 10.1177/1073858415591964 26150316

[B53] Follett KA, Weaver FM, Stern M, Hur K, Harris CL, Luo P, Marks WJ Jr, Rothlind J, Sagher O, Moy C, Pahwa R, Burchiel K, Hogarth P, Lai EC, Duda JE, Holloway K, Samii A, Horn S, Bronstein JM, Stoner G, et al. (2010) Pallidal versus subthalamic deep-brain stimulation for Parkinson's disease. N Engl J Med 362:2077–2091. 10.1056/NEJMoa0907083 20519680

[B54] François C, Savy C, Jan C, Tande D, Hirsch EC, Yelnik J (2000) Dopaminergic innervation of the subthalamic nucleus in the normal state, in MPTP-treated monkeys, and in Parkinson's disease patients. J Comp Neur 425:121–129. 10940946 10.1002/1096-9861(20000911)425:1<121::aid-cne10>3.0.co;2-g

[B55] Frank MJ, Samanta J, Moustafa AA, Sherman SJ (2007) Hold your horses: impulsivity, deep brain stimulation, and medication in parkinsonism. Science 318:1309–1312. 10.1126/science.1146157 17962524

[B56] Giannicola G, Marceglia S, Rossi L, Mrakic-Sposta S, Rampini P, Tamma F, Cogiamanian F, Barbieri S, Priori A (2010) The effects of levodopa and ongoing deep brain stimulation on subthalamic beta oscillations in Parkinson's disease. Exp Neurol 226:120–127. 10.1016/j.expneurol.2010.08.011 20713047

[B57] Gradinaru V, Mogri M, Thompson KR, Henderson JM, Deisseroth K (2009) Optical deconstruction of parkinsonian neural circuitry. Science 324:354–359. 10.1126/science.1167093 19299587 PMC6744370

[B58] Hälbig TD, Tse W, Frisina PG, Baker BR, Hollander E, Shapiro H, Tagliati M, Koller WC, Olanow CW (2009) Subthalamic deep brain stimulation and impulse control in Parkinson's disease. Eur J Neurol 16:493–497. 10.1111/j.1468-1331.2008.02509.x 19236471

[B59] Hamada I, DeLong MR (1992) Excitotoxic acid lesions of the primate subthalamic nucleus result in transient dyskinesias of the contralateral limbs. J Neurophysiol 68:1850–1858. 1479448 10.1152/jn.1992.68.5.1850

[B60] Hamani C, Temel Y (2012) Deep brain stimulation for psychiatric disease: contributions and validity of animal models. Sci Transl Med 4:142rv8. 10.1126/scitranslmed.3003722 22786683

[B61] Hamani C, Saint-Cyr JA, Fraser J, Kaplitt M, Lozano AM (2004) The subthalamic nucleus in the context of movement disorders. Brain 127:4–20. 10.1093/brain/awh029 14607789

[B62] Hamani C, Machado DC, Hipólide DC, Dubiela FP, Suchecki D, Macedo CE, Tescarollo F, Martins U, Covolan L, Nobrega JN (2012) Deep brain stimulation reverses anhedonic-like behavior in a chronic model of depression: role of serotonin and brain derived neurotrophic factor. Biol Psychiatry 71:30–35. 10.1016/j.biopsych.2011.08.025 22000731 PMC5756076

[B63] Hammond C, Feger J, Bioulac B, Souteyrand JP (1979) Experimental hemiballism in the monkey produced by unilateral kainic acid lesion in corpus Luysii. Brain Res 171:577–580. 113048 10.1016/0006-8993(79)91066-7

[B64] Hashimoto T, Elder CM, Okun MS, Patrick SK, Vitek JL (2003) Stimulation of the subthalamic nucleus changes the firing pattern of pallidal neurons. J Neurosci 23:1916–1923. 12629196 10.1523/JNEUROSCI.23-05-01916.2003 PMC6741976

[B65] Hassani OK, Mouroux M, Féger J (1996) Increased subthalamic neuronal activity after nigral dopaminergic lesion independent of disinhibition via the globus pallidus. Neuroscience 72:105–115. 8730710 10.1016/0306-4522(95)00535-8

[B66] Haynes WI, Mallet L (2010) High-frequency stimulation of deep brain structures in obsessive-compulsive disorder: the search for a valid circuit. Eur J Neurosci 32:1118–1127. 10.1111/j.1460-9568.2010.07418.x 21039951

[B67] Haynes WI, Haber SN (2013) The organization of prefrontal-subthalamic inputs in primates provides an anatomical substrate for both functional specificity and integration: implications for Basal Ganglia models and deep brain stimulation. J Neurosci 33:4804–4814. 10.1523/JNEUROSCI.4674-12.2013 23486951 PMC3755746

[B68] Heinsen H, Arzberger T, Schmitz C (2000) Celloidin mounting (embedding without infiltration) - a new, simple and reliable method for producing serial sections of high thickness through complete human brains and its application to stereological and immunohistochemical investigations. J Chem Neuroanat 20:49–59. 11074343 10.1016/s0891-0618(00)00067-3

[B69] Herzog J, Fietzek U, Hamel W, Morsnowski A, Steigerwald F, Schrader B, Weinert D, Pfister G, Müller D, Mehdorn HM, Deuschl G, Volkmann J (2004) Most effective stimulation site in subthalamic deep brain stimulation for Parkinson's disease. Mov Disord 19:1050–1054. 10.1002/mds.20056 15372594

[B70] Hutchison WD, Allan RJ, Opitz H, Levy R, Dostrovsky JO, Lang AE, Lozano AM (1998) Neurophysiological identification of the subthalamic nucleus in surgery for Parkinson's disease. Ann Neurol 44:622–628. 10.1002/ana.410440407 9778260

[B71] Joel D (2006) Current animal models of obsessive compulsive disorder: a critical review. Prog Neuropsychopharmacol Biol Psychiatry 30:374–388. 10.1016/j.pnpbp.2005.11.006 16457927

[B72] Johnson LA, Nebeck SD, Muralidharan A, Johnson MD, Baker KB, Vitek JL (2016) Closed-loop deep brain stimulation effects on Parkinsonian motor symptoms in a non-human primate - is beta enough? Brain Stimul 9:892–896. 10.1016/j.brs.2016.06.051 27401045 PMC5143196

[B73] Kass JI, Mintz IM (2006) Silent plateau potentials, rhythmic bursts, and pacemaker firing: three patterns of activity that coexist in quadristable subthalamic neurons. Proc Natl Acad Sci USA 103:183–188. 10.1073/pnas.0506781102 16373507 PMC1324981

[B74] Kilgore KL, Bhadra N (2004) Nerve conduction block utilising high-frequency alternating current. Med Biol Eng Comput 42:394–406. 15191086 10.1007/BF02344716

[B75] Kim R, Nakano K, Jayaraman A, Carpenter MB (1976) Projections of the globus pallidus and adjacent structures: an autoradiographic study in the monkey. J Comp Neur 169:263–290. 10.1002/cne.901690302 823180

[B76] Klavir O, Flash S, Winter C, Joel D (2009) High frequency stimulation and pharmacological inactivation of the subthalamic nucleus reduces 'compulsive' lever-pressing in rats. Exp Neurol 215:101–109. 10.1016/j.expneurol.2008.09.017 18951894

[B77] Krack P, Hariz MI, Baunez C, Guridi J, Obeso JA (2010) Deep brain stimulation: from neurology to psychiatry? Trends Neurosci 33:474–484. 10.1016/j.tins.2010.07.002 20832128

[B78] Kringelbach ML, Jenkinson N, Owen SL, Aziz TZ (2007) Translational principles of deep brain stimulation. Nat Rev Neurosci 8:623–635. 10.1038/nrn2196 17637800

[B79] Kühn AA, Kempf F, Büucke C, Gaynor Doyle L, Martinez-Torres I, Pogosyan A, Trottenberg T, Kupsch A, Schneider GH, Hariz MI, Vandenberghe W, Nuttin B, Brown P (2008) High-frequency stimulation of the subthalamic nucleus suppresses oscillatory beta activity in patients with Parkinson's disease in parallel with improvement in motor performance. J Neurosci 28:6165–6173. 10.1523/JNEUROSCI.0282-08.2008 18550758 PMC6670522

[B80] Kühn AA, Tsui A, Aziz T, Ray N, Brücke C, Kupsch A, Schneider GH, Brown P (2009) Pathological synchronisation in the subthalamic nucleus of patients with Parkinson's disease relates to both bradykinesia and rigidity. Exp Neurol 215:380–387. 10.1016/j.expneurol.2008.11.008 19070616

[B81] Kuo JS, Carpenter MB (1973) Organization of pallidothalamic projections in the rhesus monkey. J Comp Neur 151:201–236. 10.1002/cne.901510302 4126710

[B82] Lambert C, Zrinzo L, Nagy Z, Lutti A, Hariz M, Foltynie T, Draganski B, Ashburner J, Frackowiak R (2012) Confirmation of functional zones within the human subthalamic nucleus: patterns of connectivity and sub-parcellation using diffusion weighted imaging. Neuroimage 60:83–94. 10.1016/j.neuroimage.2011.11.082 22173294 PMC3315017

[B83] Lardeux S, Baunez C (2008) Alcohol preference influences the subthalamic nucleus control on motivation for alcohol in rats. Neuropsychopharmacology 33:634–642. 10.1038/sj.npp.1301432 17460610

[B84] Lavoie B, Parent A (1994) Pedunculopontine nucleus in the squirrel monkey: projections to the basal ganglia as revealed by anterograde tract-tracing methods. J Comp Neur 344:210–231. 10.1002/cne.903440204 8077458

[B85] Lavoie B, Smith Y, Parent A (1989) Dopaminergic innervation of the basal ganglia in the squirrel monkey as revealed by tyrosine hydroxylase immunohistochemistry. J Comp Neur 289:36–52. 10.1002/cne.902890104 2572613

[B86] Lee MS, Marsden CD (1994) Movement disorders following lesions of the thalamus or subthalamic region. Mov Disord 9:493–507. 10.1002/mds.870090502 7990845

[B87] Lévesque JC, Parent A (2005) GABAergic interneurons in human subthalamic nucleus. Mov Disord 20:574–584. 10.1002/mds.20374 15645534

[B88] Levy R, Hutchison WD, Lozano AM, Dostrovsky JO (2000) High-frequency synchronization of neuronal activity in the subthalamic nucleus of parkinsonian patients with limb tremor. J Neurosci 20:7766–7775. 11027240 10.1523/JNEUROSCI.20-20-07766.2000 PMC6772896

[B89] Levy R, Ashby P, Hutchison WD, Lang AE, Lozano AM, Dostrovsky JO (2002) Dependence of subthalamic nucleus oscillations on movement and dopamine in Parkinson's disease. Brain 125:1196–1209. 12023310 10.1093/brain/awf128

[B90] Limousin P, Pollak P, Benazzouz A, Hoffmann D, Le Bas JF, Broussolle E, Perret JE, Benabid AL (1995) Effect of parkinsonian signs and symptoms of bilateral subthalamic nucleus stimulation. Lancet 345:91–95. 7815888 10.1016/s0140-6736(95)90062-4

[B91] Little S, Pogosyan A, Neal S, Zavala B, Zrinzo L, Hariz M, Foltynie T, Limousin P, Ashkan K, FitzGerald J, Green AL, Aziz TZ, Brown P (2013) Adaptive deep brain stimulation in advanced Parkinson disease. Ann Neurol 74:449–457. 10.1002/ana.23951 23852650 PMC3886292

[B92] López-Azcárate J, Tainta M, Rodríguez-Oroz MC, Valencia M, González R, Guridi J, Iriarte J, Obeso JA, Artieda J, Alegre M (2010) Coupling between beta and high-frequency activity in the human subthalamic nucleus may be a pathophysiological mechanism in Parkinson's disease. J Neurosci 30:6667–6677. 10.1523/JNEUROSCI.5459-09.2010 20463229 PMC6632566

[B93] Lozano AM, Dostrovsky J, Chen R, Ashby P (2002) Deep brain stimulation for Parkinson's disease: disrupting the disruption. Lancet Neurol 1:225–231. 12849455 10.1016/s1474-4422(02)00101-1

[B94] Magariños-Ascone C, Pazo JH, Macadar O, Buño W (2002) High-frequency stimulation of the subthalamic nucleus silences subthalamic neurons: a possible cellular mechanism in Parkinson's disease. Neuroscience 115:1109–1117. 12453483 10.1016/s0306-4522(02)00538-9

[B95] Magariños-Ascone CM, Figueiras-Mendez R, Riva-Meana C, Córdoba-Fernández A (2000) Subthalamic neuron activity related to tremor and movement in Parkinson's disease. Eur J Neurosci 12:2597–2607. 10947834 10.1046/j.1460-9568.2000.00127.x

[B96] Magill PJ, Bolam JP, Bevan MD (2001) Dopamine regulates the impact of the cerebral cortex on the subthalamic nucleus-globus pallidus network. Neuroscience 106:313–330. 11566503 10.1016/s0306-4522(01)00281-0

[B97] Mallet L, Mesnage V, Houeto JL, Pelissolo A, Yelnik J, Behar C, Gargiulo M, Welter ML, Bonnet AM, Pillon B, Cornu P, Dormont D, Pidoux B, Allilaire JF, Agid Y (2002) Compulsions, Parkinson's disease, and stimulation. Lancet 360:1302–1304. 10.1016/S0140-6736(02)11339-0 12414208

[B98] Mallet L, Polosan M, Jaafari N, Baup N, Welter ML, Fontaine D, du Montcel ST, Yelnik J, Chéreau I, Arbus C, Raoul S, Aouizerate B, Damier P, Chabardès S, Czernecki V, Ardouin C, Krebs MO, Bardinet E, Chaynes P, Burbaud P, et al. (2008a) Subthalamic nucleus stimulation in severe obsessive-compulsive disorder. N Engl J Med 359:2121–2134. 10.1056/NEJMoa0708514 19005196

[B99] Mallet N, Pogosyan A, Sharott A, Csicsvari J, Bolam JP, Brown P, Magill PJ (2008b) Disrupted dopamine transmission and the emergence of exaggerated beta oscillations in subthalamic nucleus and cerebral cortex. J Neurosci 28:4795–4806. 18448656 10.1523/JNEUROSCI.0123-08.2008 PMC6670450

[B100] Matsumura M, Kojima J, Gardiner TW, Hikosaka O (1992) Visual and oculomotor functions of monkey subthalamic nucleus. J Neurophysiol 67:1615–1632. 1629767 10.1152/jn.1992.67.6.1615

[B101] Maurice N, Thierry AM, Glowinski J, Deniau JM (2003) Spontaneous and evoked activity of substantia nigra pars reticulata neurons during high-frequency stimulation of the subthalamic nucleus. J Neurosci 23:9929–9936. 14586023 10.1523/JNEUROSCI.23-30-09929.2003 PMC6740874

[B102] Meissner W, Leblois A, Hansel D, Bioulac B, Gross CE, Benazzouz A, Boraud T (2005) Subthalamic high frequency stimulation resets subthalamic firing and reduces abnormal oscillations. Brain 128:2372–2382. 10.1093/brain/awh616 16123144

[B103] Mesulam MM, Mash D, Hersh L, Bothwell M, Geula C (1992) Cholinergic innervation of the human striatum, globus pallidus, subthalamic nucleus, substantia nigra, and red nucleus. J Comp Neur 323:252–268. 10.1002/cne.903230209 1401259

[B104] Nambu A, Takada M, Inase M, Tokuno H (1996) Dual somatotopical representations in the primate subthalamic nucleus: evidence for ordered but reversed body-map transformations from the primary motor cortex and the supplementary motor area. J Neurosci 16:2671–2683. 8786443 10.1523/JNEUROSCI.16-08-02671.1996 PMC6578767

[B105] Nambu A, Tokuno H, Inase M, Takada M (1997) Corticosubthalamic input zones from forelimb representations of the dorsal and ventral divisions of the premotor cortex in the macaque monkey: comparison with the input zones from the primary motor cortex and the supplementary motor area. Neurosci Lett 239:13–16. 9547160 10.1016/s0304-3940(97)00877-x

[B106] Nambu A, Tokuno H, Hamada I, Kita H, Imanishi M, Akazawa T, Ikeuchi Y, Hasegawa N (2000) Excitatory cortical inputs to pallidal neurons via the subthalamic nucleus in the monkey. J Neurophysiol 84:289–300. 10899204 10.1152/jn.2000.84.1.289

[B107] O'Sullivan SS, Evans AH, Lees AJ (2009) Dopamine dysregulation syndrome: an overview of its epidemiology, mechanisms and management. CNS Drugs 23:157–170. 19173374 10.2165/00023210-200923020-00005

[B108] Okun MS, Fernandez HH, Wu SS, Kirsch-Darrow L, Bowers D, Bova F, Suelter M, Jacobson CE 4th, Wang X, Gordon CW Jr, Zeilman P, Romrell J, Martin P, Ward H, Rodriguez RL, Foote KD (2009) Cognition and mood in Parkinson's disease in subthalamic nucleus versus globus pallidus interna deep brain stimulation: the COMPARE trial. Ann Neurol 65:586–595. 10.1002/ana.21596 19288469 PMC2692580

[B109] Parent A, Hazrati LN (1993) Anatomical aspects of information processing in primate basal ganglia. Trends Neurosci 16:111–116. 7681234 10.1016/0166-2236(93)90135-9

[B110] Parent A, Hazrati LN (1995a) Functional anatomy of the basal ganglia. II. The place of subthalamic nucleus and external pallidum in basal ganglia circuitry. Brain Res Brain Res Rev 20:128–154. 7711765 10.1016/0165-0173(94)00008-d

[B111] Parent A, Hazrati LN (1995b) Functional anatomy of the basal ganglia. I. The cortico-basal ganglia-thalamo-cortical loop. Brain Res Brain Res Rev 20:91–127. 7711769 10.1016/0165-0173(94)00007-c

[B112] Parent M, Parent A (2004) The pallidofugal motor fiber system in primates. Parkinsonism Relat Disord 10:203–211. 10.1016/j.parkreldis.2004.02.007 15120094

[B113] Parent M, Wallman MJ, Gagnon D, Parent A (2011) Serotonin innervation of basal ganglia in monkeys and humans. J Chem Neuroanat 41:256–265. 10.1016/j.jchemneu.2011.04.005 21664455

[B114] Périer C, Agid Y, Hirsch EC, Féger J (2000) Ipsilateral and contralateral subthalamic activity after unilateral dopaminergic lesion. Neuroreport 11:3275–3278. 11043563 10.1097/00001756-200009280-00045

[B115] Plantinga BR, Temel Y, Roebroeck A, Uludağ K, Ivanov D, Kuijf ML, Ter Haar Romenij BM (2014) Ultra-high field magnetic resonance imaging of the basal ganglia and related structures. Front Hum Neurosci 8:876 10.3389/fnhum.2014.00876 25414656 PMC4220687

[B116] Plantinga BR, Temel Y, Duchin Y, Uludag K, Patriat R, Roebroeck A, Kuijf M, Jahanshahi A, Ter Haar Romenij B, Vitek J, Harel N (2016) Individualized parcellation of the subthalamic nucleus in patients with Parkinson's disease with 7T MRI. Neuroimage pii:S1053-8119(16)30486-4. 10.1016/j.neuroimage.2016.09.023 PMC5479742 27688203

[B117] Pollo C, Vingerhoets F, Pralong E, Ghika J, Maeder P, Meuli R, Thiran JP, Villemure JG (2007) Localization of electrodes in the subthalamic nucleus on magnetic resonance imaging. J Neurosurg 106:36–44. 10.3171/jns.2007.106.1.36 17240554

[B118] Ray NJ, Jenkinson N, Wang S, Holland P, Brittain JS, Joint C, Stein JF, Aziz T (2008) Local field potential beta activity in the subthalamic nucleus of patients with Parkinson's disease is associated with improvements in bradykinesia after dopamine and deep brain stimulation. Exp Neurol 213:108–113. 10.1016/j.expneurol.2008.05.008 18619592

[B119] Robledo P, Féger J (1990) Excitatory influence of rat subthalamic nucleus to substantia nigra pars reticulata and the pallidal complex: electrophysiological data. Brain Res 518:47–54. 2390727 10.1016/0006-8993(90)90952-8

[B120] Rodriguez MC, Obeso JA, Olanow CW (1998) Subthalamic nucleus-mediated excitotoxicity in Parkinson's disease: a target for neuroprotection. Ann Neurol 44:S175–S188. 9749591 10.1002/ana.410440726

[B121] Rodriguez-Oroz MC, Obeso JA, Lang AE, Houeto JL, Pollak P, Rehncrona S, Kulisevsky J, Albanese A, Volkmann J, Hariz MI, Quinn NP, Speelman JD, Guridi J, Zamarbide I, Gironell A, Molet J, Pascual-Sedano B, Pidoux B, Bonnet AM, Agid Y, et al. (2005) Bilateral deep brain stimulation in Parkinson's disease: a multicentre study with 4 years follow-up. Brain 128:2240–2249. 10.1093/brain/awh571 15975946

[B122] Rosin B, Slovik M, Mitelman R, Rivlin-Etzion M, Haber SN, Israel Z, Vaadia E, Bergman H (2011) Closed-loop deep brain stimulation is superior in ameliorating parkinsonism. Neuron 72:370–384. 10.1016/j.neuron.2011.08.023 22017994

[B123] Rothlind JC, York MK, Carlson K, Luo P, Marks WJ Jr, Weaver FM, Stern M, Follett K, Reda D; CSP-468 Study Group (2015) Neuropsychological changes following deep brain stimulation surgery for Parkinson's disease: comparisons of treatment at pallidal and subthalamic targets versus best medical therapy. J Neurol Neurosurg Psychiatry 86:622–629. 10.1136/jnnp-2014-308119 25185211

[B124] Rouaud T, Lardeux S, Panayotis N, Paleressompoulle D, Cador M, Baunez C (2010) Reducing the desire for cocaine with subthalamic nucleus deep brain stimulation. Proc Natl Acad Sci USA 107:1196–1200. 10.1073/pnas.0908189107 20080543 PMC2824319

[B125] Sadikot AF, Parent A, François C (1992) Efferent connections of the centromedian and parafascicular thalamic nuclei in the squirrel monkey: a PHA-L study of subcortical projections. J Comp Neur 315:137–159. 10.1002/cne.903150203 1372010

[B126] Shimamoto SA, Ryapolova-Webb ES, Ostrem JL, Galifianakis NB, Miller KJ, Starr PA (2013) Subthalamic nucleus neurons are synchronized to primary motor cortex local field potentials in Parkinson's disease. J Neurosci 33:7220–7233. 10.1523/JNEUROSCI.4676-12.2013 23616531 PMC3673303

[B127] Shink E, Bevan MD, Bolam JP, Smith Y (1996) The subthalamic nucleus and the external pallidum: two tightly interconnected structures that control the output of the basal ganglia in the monkey. Neuroscience 73:335–357. 8783253 10.1016/0306-4522(96)00022-x

[B128] Smith Y, Hazrati LN, Parent A (1990) Efferent projections of the subthalamic nucleus in the squirrel monkey as studied by the PHA-L anterograde tracing method. J Comp Neur 294:306–323. 10.1002/cne.902940213 2332533

[B129] Syed EC, Benazzouz A, Taillade M, Baufreton J, Champeaux K, Falgairolle M, Bioulac B, Gross CE, Boraud T (2012) Oscillatory entrainment of subthalamic nucleus neurons and behavioural consequences in rodents and primates. Eur J Neurosci 36:3246–3257. 10.1111/j.1460-9568.2012.08246.x 22853738

[B130] Tai CH, Boraud T, Bezard E, Bioulac B, Gross C, Benazzouz A (2003) Electrophysiological and metabolic evidence that high-frequency stimulation of the subthalamic nucleus bridles neuronal activity in the subthalamic nucleus and the substantia nigra reticulata. FASEB J 17:1820–1830. 10.1096/fj.03-0163com 14519661

[B131] Tass PA, Qin L, Hauptmann C, Dovero S, Bezard E, Boraud T, Meissner WG (2012) Coordinated reset has sustained aftereffects in Parkinsonian monkeys. Ann Neurol 72:816–820. 10.1002/ana.23663 23280797

[B132] Temel Y, Visser-Vandewalle V, Aendekerk B, Rutten B, Tan S, Scholtissen B, Schmitz C, Blokland A, Steinbusch HW (2005) Acute and separate modulation of motor and cognitive performance in parkinsonian rats by bilateral stimulation of the subthalamic nucleus. Exp Neurol 193:43–52. 10.1016/j.expneurol.2004.12.025 15817263

[B133] Temel Y, Blokland A, Ackermans L, Boon P, van Kranen-Mastenbroek VH, Beuls EA, Spincemaille GH, Visser-Vandewalle V (2006a) Differential effects of subthalamic nucleus stimulation in advanced Parkinson disease on reaction time performance. Exp Brain Res 169:389–399. 16273395 10.1007/s00221-005-0151-6

[B134] Temel Y, Kessels A, Tan S, Topdag A, Boon P, Visser-Vandewalle V (2006b) Behavioural changes after bilateral subthalamic stimulation in advanced Parkinson disease: a systematic review. Parkinsonism Relat Disord 12:265–272. 16621661 10.1016/j.parkreldis.2006.01.004

[B135] Temel Y, Boothman LJ, Blokland A, Magill PJ, Steinbusch HW, Visser-Vandewalle V, Sharp T (2007) Inhibition of 5-HT neuron activity and induction of depressive-like behavior by high-frequency stimulation of the subthalamic nucleus. Proc Natl Acad Sci USA 104:17087–17092. 10.1073/pnas.0704144104 17942692 PMC2040465

[B136] Tournier JD, Calamante F, Connelly A (2007) Robust determination of the fibre orientation distribution in diffusion MRI: non-negativity constrained super-resolved spherical deconvolution. Neuroimage 35:1459–1472. 10.1016/j.neuroimage.2007.02.016 17379540

[B137] Uslaner JM, Robinson TE (2006) Subthalamic nucleus lesions increase impulsive action and decrease impulsive choice - mediation by enhanced incentive motivation? Eur J Neurosci 24:2345–2354. 10.1111/j.1460-9568.2006.05117.x 17074055

[B138] Vitek JL (2002) Mechanisms of deep brain stimulation: excitation or inhibition. Mov Disord 17 [Suppl3]:S69–S72. 11948757 10.1002/mds.10144

[B139] Weaver FM, Follett K, Stern M, Hur K, Harris C, Marks WJ Jr, Rothlind J, Sagher O, Reda D, Moy CS, Pahwa R, Burchiel K, Hogarth P, Lai EC, Duda JE, Holloway K, Samii A, Horn S, Bronstein J, Stoner G, et al. (2009) Bilateral deep brain stimulation vs best medical therapy for patients with advanced Parkinson disease: a randomized controlled trial. JAMA 301:63–73. 19126811 10.1001/jama.2008.929 PMC2814800

[B140] Wichmann T, Bergman H, DeLong MR (1994a) The primate subthalamic nucleus. I. Functional properties in intact animals. J Neurophysiol 72:494–506. 7983514 10.1152/jn.1994.72.2.494

[B141] Wichmann T, Bergman H, DeLong MR (1994b) The primate subthalamic nucleus. III. Changes in motor behavior and neuronal activity in the internal pallidum induced by subthalamic inactivation in the MPTP model of parkinsonism. J Neurophysiol 72:521–530. 7983516 10.1152/jn.1994.72.2.521

[B142] Windels F, Bruet N, Poupard A, Urbain N, Chouvet G, Feuerstein C, Savasta M (2000) Effects of high frequency stimulation of subthalamic nucleus on extracellular glutamate and GABA in substantia nigra and globus pallidus in the normal rat. Eur J Neurosci 12:4141–4146. 11069610 10.1046/j.1460-9568.2000.00296.x

[B143] Windels F, Carcenac C, Poupard A, Savasta M (2005) Pallidal origin of GABA release within the substantia nigra pars reticulata during high-frequency stimulation of the subthalamic nucleus. J Neurosci 25:5079–5086. 10.1523/JNEUROSCI.0360-05.2005 15901790 PMC6724863

[B144] Wingeier B, Tcheng T, Koop MM, Hill BC, Heit G, Bronte-Stewart HM (2006) Intra-operative STN DBS attenuates the prominent beta rhythm in the STN in Parkinson's disease. Exp Neurol 197:244–251. 10.1016/j.expneurol.2005.09.016 16289053

[B145] Winstanley CA (2011) The utility of rat models of impulsivity in developing pharmacotherapies for impulse control disorders. Br J Pharmacol 164:1301–1321. 10.1111/j.1476-5381.2011.01323.x 21410459 PMC3229763

[B146] Winstanley CA, Baunez C, Theobald DE, Robbins TW (2005) Lesions to the subthalamic nucleus decrease impulsive choice but impair autoshaping in rats: the importance of the basal ganglia in Pavlovian conditioning and impulse control. Eur J Neurosci 21:3107–3116. 10.1111/j.1460-9568.2005.04143.x 15978020

[B147] Winter C, Mundt A, Jalali R, Joel D, Harnack D, Morgenstern R, Juckel G, Kupsch A (2008) High frequency stimulation and temporary inactivation of the subthalamic nucleus reduce quinpirole-induced compulsive checking behavior in rats. Exp Neurol 210:217–228. 10.1016/j.expneurol.2007.10.020 18076877

[B148] Wu XX, Shuai JW (2012) Multistability in a neuron model with extracellular potassium dynamics. Phys Rev E Stat Nonlin Soft Matter Phys 85:061911. 10.1103/PhysRevE.85.061911 23005131

[B149] Zheng F, Lammert K, Nixdorf-Bergweiler BE, Steigerwald F, Volkmann J, Alzheimer C (2011) Axonal failure during high frequency stimulation of rat subthalamic nucleus. J Physiol 589:2781–2793. 10.1113/jphysiol.2011.205807 21486784 PMC3112555

